# Preclinical evaluation of the anti-tumor effects of the natural isoflavone genistein in two xenograft mouse models monitored by [^18^F]FDG, [^18^F]FLT, and [^64^Cu]NODAGA-cetuximab small animal PET

**DOI:** 10.18632/oncotarget.8625

**Published:** 2016-04-07

**Authors:** Valerie S. Honndorf, Stefan Wiehr, Anna-Maria Rolle, Julia Schmitt, Luisa Kreft, Letitia Quintanilla-Martinez, Ursula Kohlhofer, Gerald Reischl, Andreas Maurer, Karsten Boldt, Michael Schwarz, Holger Schmidt, Bernd J. Pichler

**Affiliations:** ^1^ Werner Siemens Imaging Center, Department of Preclinical Imaging and Radiopharmacy, Eberhard Karls University, Tuebingen, Germany; ^2^ Institute of Pathology, University Hospital, Eberhard Karls University, Tuebingen, Germany; ^3^ Medical Proteome Center, Institute for Ophthalmic Research, Eberhard Karls University, Tuebingen, Germany; ^4^ Institute of Experimental and Clinical Pharmacology and Toxicology, Department of Toxicology, Eberhard Karls University, Tuebingen, Germany; ^5^ Department of Radiology, Diagnostic and Interventional Radiology, Eberhard Karls University, Tuebingen, Germany

**Keywords:** PET, genistein and cetuximab, A431 and Colo205 xenografts, molecular tumor imaging

## Abstract

The natural phytoestrogen genistein is known as protein kinase inhibitor and tumor suppressor in various types of cancers. We studied its antitumor effect in two different xenograft models using positron emission tomography (PET) *in vivo* combined with *ex vivo* histology and nuclear magnetic resonance (NMR) metabolic fingerprinting.

**Procedures:**

A431 and Colo205 tumor-bearing mice were treated with vehicle or genistein (500 mg/kg/d) over a period of 12 days. Imaging was performed with 2-deoxy-2-[^18^F]fluoro-D-glucose ([^18^F]FDG) and 3′-deoxy-3′-[^18^F]fluorothymidine ([^18^F] FLT). In a second study A431 tumor-bearing mice were treated with vehicle, genistein (500 mg/kg/d), cetuximab (1mg/3d) or a combination of the compounds and imaged using [^18^F]FDG, [^18^F]FLT and [^64^Cu]NODAGA-cetuximab. Data were compared to histology and principal components analysis (PCA) of NMR fingerprinting data.

**Results:**

Genistein reduced tumor growth significantly in both xenografts. [^18^F] FLT uptake was consistent in both models and corresponded to histological findings and also PCA whereas [^18^F]FDG and [^64^Cu]NODAGA-cetuximab were not suitable for therapy monitoring.

**Conclusions:**

As mono-therapy the natural isoflavone genistein has a powerful therapeutic effect *in vivo* on A431 and Colo205 tumors. [^18^F]FLT has superior consistency compared to the other tested tracers in therapy monitoring, while the treatment effect could be shown on the molecular level by histology and metabolic fingerprinting.

## INTRODUCTION

Phytoestrogens are natural compounds exhibiting a similar chemical structure to that of the sexual hormone 17β-estradiol. The phytoestrogen genistein, 4′, 5, 7-trihydroxyisoflavone, is found in soybeans and soy products, and competes with 17β-estradiol for binding to the estrogen receptors ERα and ERβ inhibiting their activities and altering their mediated pathways [1]. For the past 30 years, genistein is subject of intense research showing its efficacy based on inhibitory effects on a great number of various malignancies such as prostate cancer, epidermoid carcinoma, pancreatic cancer, lung cancer, colorectal or breast cancer [2–9]. Genistein induces apoptotic cell death via G2/M cell cycle arrest, suppresses angiogenesis, and is known for its potential to inhibit signaling cascades mediated by protein tyrosine kinases [10, 11]. Experiments demonstrated that genistein inhibits the activity of the epidermal growth factor receptor (EGFR), NFκB, ERK1/2 and Akt pathways, the insulin receptor, Abl and Src kinases [12, 13]. It potentiates the therapeutic effects of EGFR inhibitors such as gefitinib, erlotinib or cetuximab, which was also shown in preclinical studies [2, 14, 15].

Preliminary results show that genistein could be a valid alternative to other chemotherapeutic drugs for anticancer applications. Until now, there are still a large number of tyrosine kinase phosphorylation cascades modulated by genistein that are not fully understood.

Non-invasive positron emission tomography (PET) makes use of radiolabeled tracers to obtain molecular information of tissue specific characteristics. The standard PET tracer for tumor imaging is the glucose analogue 2-deoxy-2-[^18^F]fluoro-D-glucose ([^18^F]FDG) [16]. Malignant cells exhibit an accelerated glucose metabolism due to an up-regulation of glucose transporters and hexokinase activity, known as the Warburg effect. [^18^F]FDG accumulates in these cells leading to the differentiation of malignant and non-malignant tissue through increased image contrast [17]. The tracer 3′-deoxy-3′-[^18^F]-fluorothymidine ([^18^F]FLT) has often been used for tumor imaging over the last years since it is a substrate of thymidine kinase-1 (TK1) that is overexpressed in proliferating cells during the late G1 and S phases of the cell cycle when the cell synthesizes DNA [18, 19].

Radiolabeled monoclonal antibodies (mAbs) are attractive tracers for PET imaging due to their specific binding capacities to biomarkers, such as prostate specific membrane antigen (PSMA), epithelial glycoprotein-1 (eGP-1), epithelial cell adhesion molecule (EpCAM) or EGFR [20, 21]. The therapeutic mAb cetuximab can be labeled e.g. with the PET detectible isotope ^64^Cu, and is highly specific for EGFR which plays a crucial role in many cancer types.

In our study, cell culture experiments were performed to compare the effect of genistein with the synthetic derivative of 17β-estradiol ethinylestradiol. We were interested to see whether the additional kinase inhibiting properties of genistein would cause different [^18^F]FDG uptake patterns compared with ethinylestradiol which is only modulating ERα and ERβ activity.

Furthermore, we monitored the impact of genistein therapy using the two PET tracers [^18^F]FDG and [^18^F] FLT *in vivo* in two xenograft mouse models bearing the EGFR overexpressing epidermoid carcinoma A431 or the colorectal cancer Colo205, respectively, and compared it with histology. Additionally, we chose the A431 xenograft model for PET imaging using the radiolabeled mAb [^64^Cu]NODAGA-cetuximab because the cell line exhibits a much higher expression of EGFR compared to Colo205 [22, 23]. Aside from [^18^F]FDG and [^18^F] FLT, the radiolabeled mAb [^64^Cu]NODAGA-cetuximab could possibly detect the inhibitory effect of genistein on EGFR. Besides genistein, cetuximab as well as a combination therapy of genistein and cetuximab were used as treatments. The impact of the three different therapeutic strategies was further analyzed by Western Blot and *ex vivo* nuclear magnetic resonance (NMR)-based metabolic fingerprinting.

## RESULTS

### Genistein treatment in A431 and Colo205 xenografts

#### Cell culture

**Figure 1:**
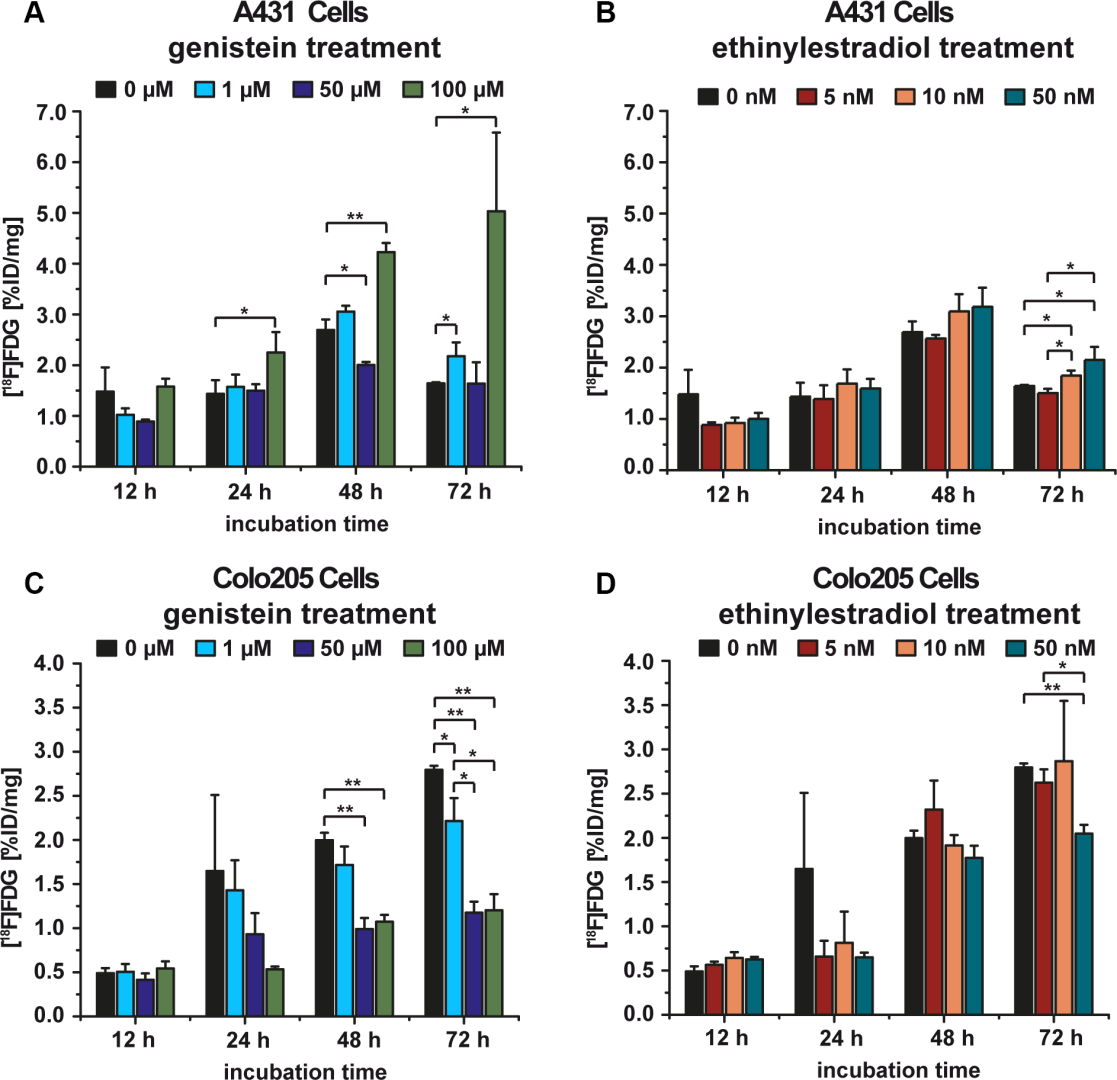
A431 and Colo205 cell culture experiments using the tracer [^18^F]FDG to monitor treatment effects of genistein or ethinylestradiol, respectively (**A**) [^18^F]FDG uptake of A431 cells after incubation with various concentrations of genistein for 12 h, 24 h, 48 h, and 72 h; (**B**) [^18^F]FDG uptake of A431 cells after incubation with different concentrations of ethinylestradiol for 12 h, 24 h, 48 h, and 72 h; (**C**) [^18^F]FDG uptake of Colo205 cells after incubation with different concentrations of genistein for 12 h, 24 h, 48 h, and 72 h; (**D**) [^18^F]FDG uptake of Colo205 cells after incubation with various concentrations of ethinylestradiol for 12 h, 24 h, 48 h, and 72 h. Data are shown as mean ± standard deviation (SD).

For the cell culture experiment the [^18^F]FDG uptake of A431 and Colo205 cells was tested after exposure to various concentrations of genistein (0 μM, 1 μM, 50 μM, 100 μM) for 12 h, 24 h, 48 h or 72 h. Additionally, to examine if the tracer uptake would be influenced by ethinylestradiol, in a second experiment both A431 and Colo205 cells were incubated with various concentrations of ethinylestradiol (0 nM, 5 nM, 10 nM, 50 nM) using the same incubation times as for the genistein treatment. The [^18^F]FDG uptake (%ID/mg protein) of A431 cells incubated with genistein is shown in Figure 1A. For each time point the highest concentration of genistein caused the highest [^18^F]FDG uptake. After 24 h, 48 h and 72 h a significant difference between 100 μM genistein and the control cells (*p* = 0.04, *p* = 0.0006 and *p* = 0.02) was found. The 50 μM genistein treatment caused a significantly lower tracer uptake after 48 h compared to the control cells (*p* = 0.005). After 72 h there was a significantly higher tracer uptake of the cells treated with 50 nM and 10 nM ethinylestradiol compared to the control group (*p* = 0.03 and *p* = 0.03, respectively) as well as to the 5 nM ethinylestradiol group (*p* = 0.01 and *p* = 0.01, respectively) whereas no significant differences were found at any other time point or concentration compared to the control cells (Figure 1B).

The cell culture experiment using Colo205 cells showed the significantly lowest tracer uptake for a treatment with 100 μM genistein compared to the control at 48 h and 72 h (*p* = 0.0001 and *p* = 0.0001, respectively). Additionally, there was a significantly lower uptake for 50 μM genistein at 48 h and 72 h (*p* = 0.0003 and *p* = 0.00003, respectively) as well as for 1μM genistein at 72 h (*p* = 0.02) compared to the control (Figure 1C). For the cells incubated with 50 nM ethinylestradiol, the tracer uptake revealed to be significantly lower compared to the control group at 72 h (0.0003), whereas no changes in tracer uptake compared to the control were found at any other time point (Figure 1D).

#### Tumor growth

**Figure 2:**
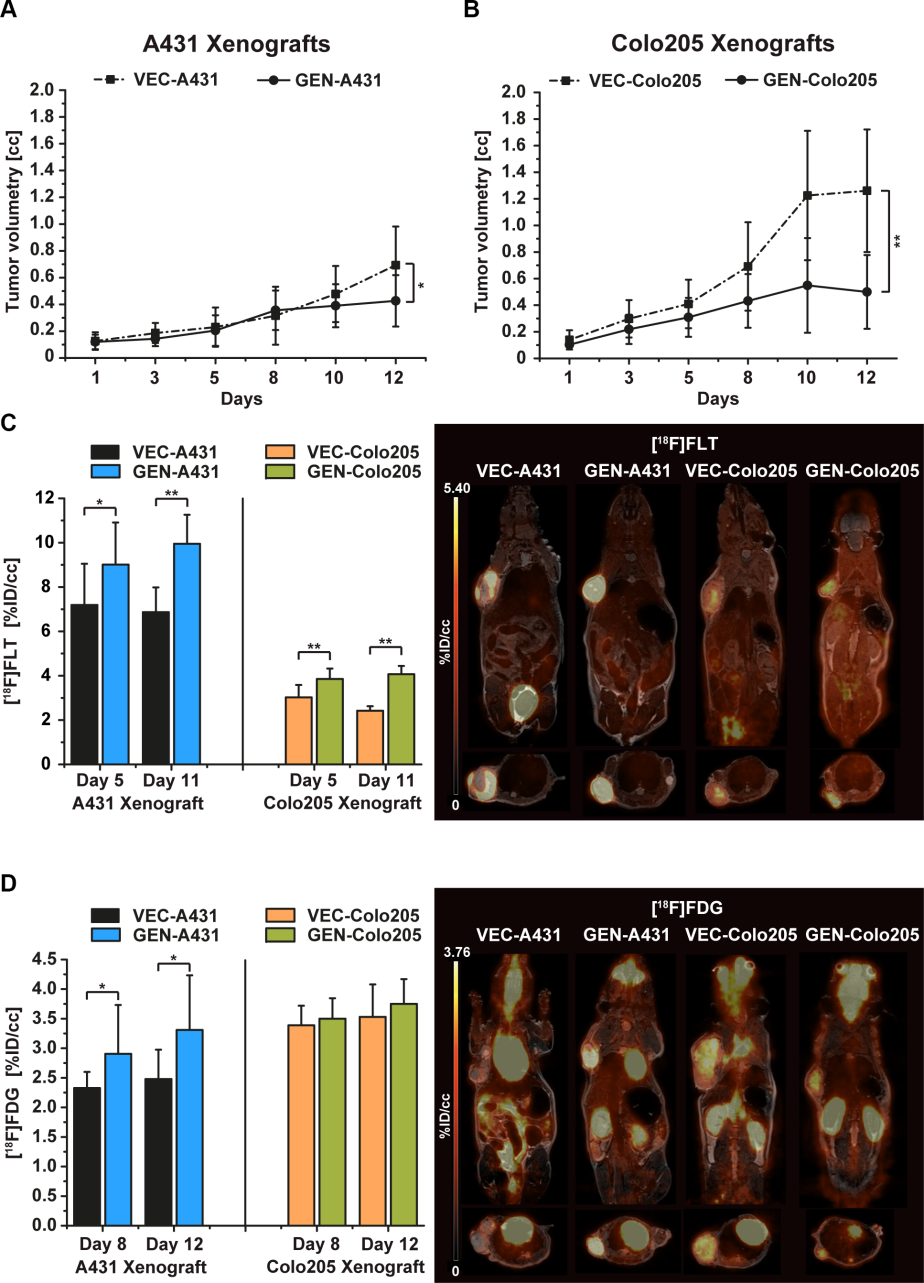
Tumor growth of vehicle-treated and genistein-treated A431 and Colo205 xenografts, and [^18^F]FLT as well as [^18^F]FDG tumor uptake monitoring the treatment effects (**A**) Tumor growth of the A431 tumor xenografts revealed significantly smaller tumor volumes of genistein-treated animals (GEN-A431) compared to vehicle-treated (VEC-A431, *p* = 0.03) over a treatment period of 12 days. (**B**) Tumor growth of the Colo205 tumor xenografts revealed significantly smaller tumor volumes of genistein-treated animals (GEN-Colo205) compared to vehicle-treated (VEC-Colo205, *p* = 0.0003) over a treatment period of 12 days. (**C**) [^18^F]FLT uptake of both xenograft models on days 5 and 11 of the study. In each model the genistein-treated animals revealed a significantly higher tracer uptake compared to vehicle-treated mice on both imaging days. Representative coronal and sagittal [^18^F]FLT images overlaid onto the MR anatomy are shown for A431 and Colo205 xenografts on day 11. (**D**) [^18^F]FDG uptake of both xenograft models on days 8 and 12. Significant differences in tracer uptake were found between GEN-A431 and VEC-A431 on both days. No differences were seen in the Colo205 model. Representative coronal and sagittal [^18^F]FDG images overlaid onto the MR anatomy are shown for A431 and Colo205 xenografts on day 12. Data are shown as mean ± standard deviation (SD).

The vehicle-treated group (*n* = 10) of the A431 tumor xenografts (VEC-A431) showed a constant tumor growth over the 12 days of the study (0.69 ± 0.29cc on day 12) whereas the 10 mice of the genistein-treated group (GEN-A431) revealed a significant retardation in tumor growth (0.43 ± 0.20cc, *p* = 0.03) (Figure 2A).

Tumor growth of the vehicle-treated group of Colo205 xenografts (VEC-Colo205) was extensive over the course of the study. Tumors reached a mean volume of 1.26 ± 0.46cc on day 12, compared to the genistein-treated animals (GEN-Colo205) that revealed less than half of this size (0.50 ± 0.28cc, *p* = 0.0003) (Figure 2B).

The animal weight was very stable over the whole study period, between the groups as well as the analyzed mouse cohort (see Figure S1 in the supplementary data).

#### PET imaging

Treatment was performed two hours before PET imaging for either tracer. In general, [^18^F]FLT uptake was significantly increased in treated mice compared to untreated animals in both xenograft models. The proliferation marker [^18^F]FLT was used to investigate the tumors on day 5 and day 11 of the study. In A431 xenografts the %ID/cc of [^18^F]FLT was significantly higher in genistein treated mice compared to vehicle mice on day 5 (VEC-A431 7.19 ± 1.86 and GEN-A431 9.00 ± 1.90, *p* = 0.04) and even more extensively on day 11 (VEC-A431 6.87 ± 1.12 and GEN-A431 9.95 ± 1.31, *p* = 0.00002, Figure 2C). Interestingly, the liver tissue revealed the same differences in tracer uptake on day 5 (VEC-A431 1.80 ± 0.36 and GEN-A431 2.12 ± 0.26, *p* = 0.03) and on day 11 (VEC-A431 1.78 ± 0.29 and GEN-A431 2.32 ± 0.33, *p* = 0.001) (see Figure S2A).

The uptake of [^18^F]FLT in tumors was lower in the Colo205 model compared to the A431 xenografts but nevertheless the tracer uptake was significantly higher in all tumors of genistein-treated animals on day 5 (VEC-Colo205 3.02 ± 0.56 and GEN-Colo205 3.85 ± 0.47, *p* = 0.002) as well as on day 11 (VEC-Colo205 2.42 ± 0.21 and GEN-Colo205 4.07 ± 0.37, *p* = 9.21*10^−10^) (Figure 2C). Also, the liver tissue revealed a significantly higher tracer uptake for genistein treated animals on both imaging days (on day 5: VEC-Colo205 1.88 ± 0.36 and GEN-Colo205 2.37 ± 0.53, *p* = 0.03 and on day 11: VEC-Colo205 1.67 ± 0.23 and GEN-Colo205 2.79 ± 0.55, *p* = 0.00002) (Figure S2A).

The [^18^F]FDG uptake was measured on two days of the study (day 8 and 12) to follow potential uptake changes over time (Figure 2D). On day 8, [^18^F]FDG uptake revealed to be significantly higher in the GEN-A431 group (%ID/cc 2.90 ± 0.83) compared to the VEC-A431 group (%ID/cc 2.32 ± 0.27, *p* = 0.04). The same pattern was found between the two groups on day 12 (GEN-A431 %ID/cc 3.31 ± 0.92 and VEC-A431 %ID/cc 2.47 ± 0.50 *p* = 0.02). The muscle tissue set as reference revealed no changes over time (VEC-A431 1.05 ± 0.20 on day 8 and 1.01 ± 0.20 on day 12, GEN-A431 1.02 ± 0.19 on day 8 and 1.09 ± 0.20 on day 12, Figure S2B).

Compared to the A431 xenografts, the Colo205 xenografts revealed no significant differences in tumor uptake between the two groups over time (VEC-Colo205 3.39 ± 0.33 on day 8 and 3.53 ± 0.56 on day 12, GEN-Colo205 3.50 ± 0.35 on day 8 and 3.75 ± 0.42 on day 12, Figure 2D). The muscle tissue set as reference revealed also no changes over time (VEC-Colo205 0.97 ± 0.19 on day 8 and 1.07 ± 0.25 on day 12, GEN-Colo205 0.93 ± 0.18 on day 8 and 0.99 ± 0.11 on day 12, Figure S2B).

#### Histology and immunohistochemistry

**Figure 3:**
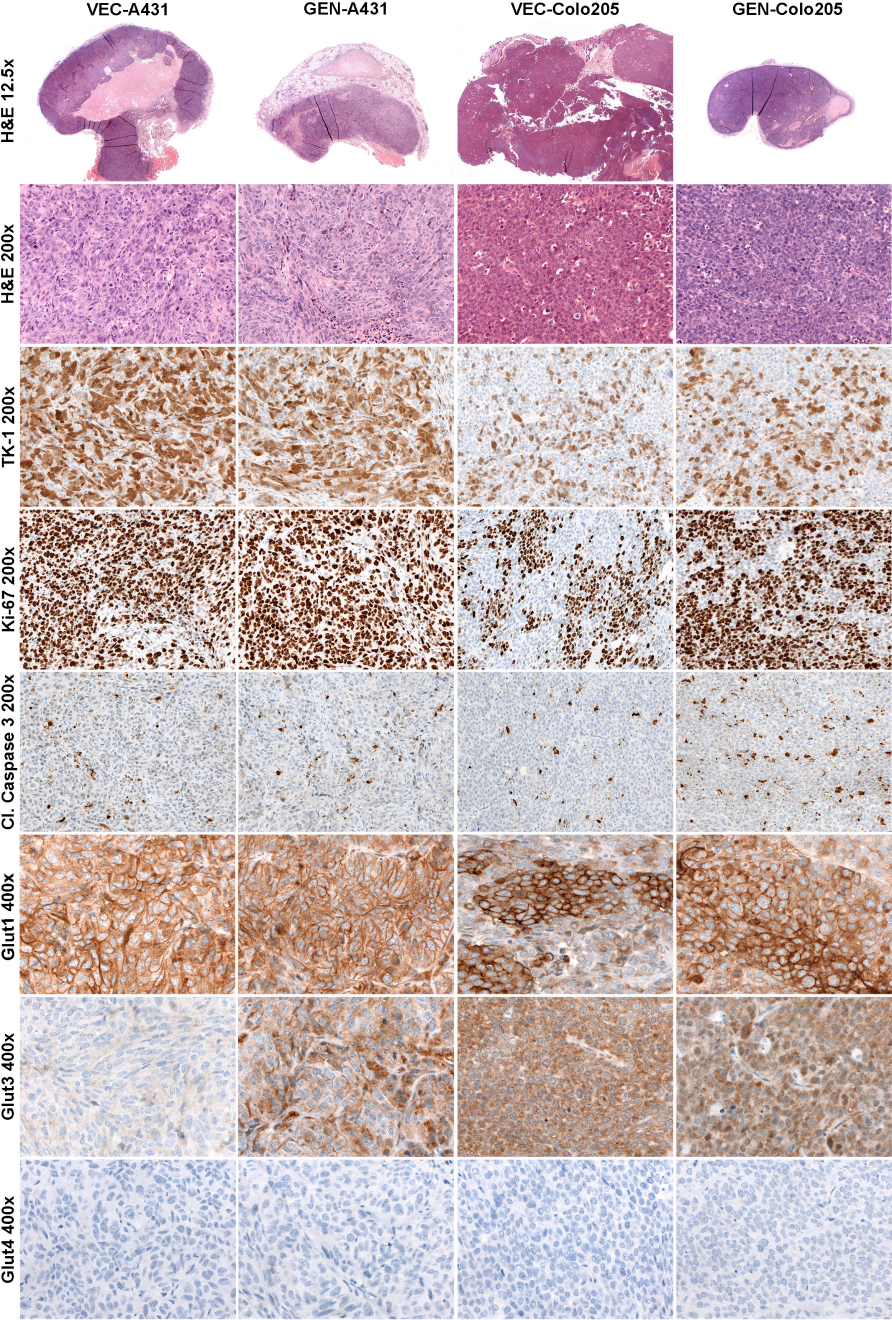
Histologically stained tumor sections of both xenograft models Hematoxylin and Eosin (H & E), thymidine kinase 1 (TK1), proliferation marker Ki67, cleaved caspase 3 and glucose transporters 1, 3 and 4. Left two columns show representative stained tumor sections of the vehicle-treated A431 (VEC-A431) and the genistein-treated A431 (GEN-A431) tumor tissues, right two columns show representative tumor sections of the vehicle-treated Colo205 (VEC-Colo205) and the genistein-treated Colo205 (GEN-Colo205) tumor tissues.

Table 1 depicts the semi-quantitative analysis of the histologically stained tumor sections of both xenograft models (representative stained sections are shown in Figure 3). Tumors of the VEC-A431 revealed significantly more necrosis compared to GEN-A431 tumors (*p* = 0.009). Besides this, significantly higher TK1 and GLUT3 indices were found for GEN-A431 tumors compared to VEC-A431 tumors (*p* = 0.002 and *p* = 0.0004, respectively). However, GLUT1, Ki67 and cleaved caspase 3 indices revealed no significant differences between both groups, and the GLUT4 index was negative for A431 tissue.

**Table 1:** Analysis of the histological tumor sections of the vehicle-treated and the genistein-treated A431 tumor mice (VEC-A431 and GEN-A431, respectively) and of the vehicle-treated and the genistein-treated Colo205 tumor mice (VEC-Colo205 and GEN-Colo205, respectively)

	VEC-A431	GEN-A431	VEC-Colo205	GEN-Colo205
	Mean [%] ± SD	Mean [%] ± SD	Mean [%] ± SD	Mean [%] ± SD
H & E	36.2 ± 9.4	19.4 ± 5.6	20.4 ± 12.3	13.7 ± 5.2
GLUT1	99.7 ± 0.7	98.6 ± 3.0	74.9 ± 7.6	94.2 ± 2.9
GLUT3	13.0 ± 9.2	67.5 ± 19.1	60.4 ± 20.5	78.0 ± 12.1
GLUT4	neg.	neg.	neg.	neg.
KI67	72.5 ± 7.9	84.2 ± 9.0	39.7 ± 13.3	76.9 ± 7.9
TK-1	50.0 ± 8.2	72.8 ± 7.8	16.8 ± 4.0	34.9 ± 4.4
Cleaved Caspase 3	11.0 ± 2.6	8.8 ± 2.0	8.4 ± 1.8	18.8 ± 2.8

The H & E results show the mean % necrosis of the tumor tissue, the immunohistochemistry results show the mean % positive cells within viable tumor regions.

Tumors of the VEC-Colo205 mice exhibited a similar amount of necrotic tissue compared to the GEN-Colo205 mice. Semi-quantitative analysis revealed that GLUT1 index was significantly higher in the GEN-Colo205 tumors (*p* = 0.001) compared to VEC-Colo205. Additionally, the cleaved caspase 3 index was significantly elevated in GEN-Colo205 (*p* = 0.0004) as well as the indices of the proliferation marker Ki67 (*p* = 0.001) and TK1 (*p* = 0.0001) compared to VEC-Colo205, whereas GLUT3 indices revealed no significant differences. The staining for GLUT4 was negative in Colo205 tumor tissue.

### Comparison of genistein, cetuximab and combined genistein-cetuximab treatments in A431 xenografts

#### Tumor growth

**Figure 4:**
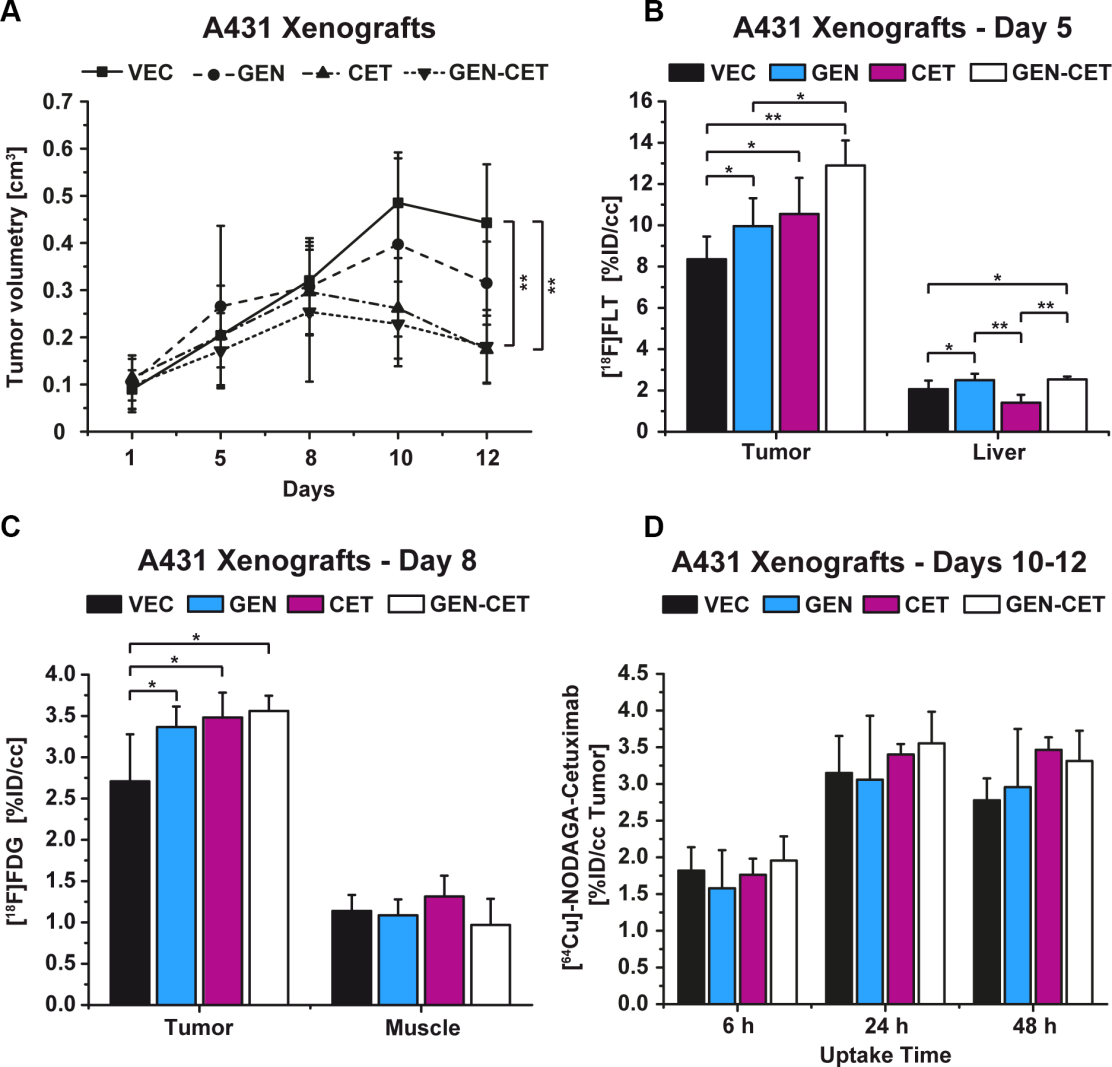
Tumor growth of vehicle-treated (VEC) and genistein-treated (GEN), cetuximab-treated (CET), and genistein-cetuximab-treated (GEN-CET) A431 xenografts, and [^18^F]FLT, [^18^F]FDG and [^64^Cu]NODAGA-cetuximab uptake, respectively, monitoring the treatment effects (**A**) Tumor growth of the four groups; significant tumor reduction was found between CET and GEN-CET compared to VEC on day 12 (*p* = 0.0003 and *p* = 0.0005, respectively). (**B**) [^18^F]FLT uptake on day 5, the GEN-CET group showed the highest tracer uptake, significantly higher than the VEC (*p* = 0.0002) and the GEN (*p* = 0.01) groups; the liver tissue revealed significantly different uptake values for each group. (**C**) [^18^F]FDG uptake on day 8. The three treatment groups showed a significantly higher tracer uptake compared to the VEC group (GEN *p* = 0.008, CET *p* = 0.004, GEN-CET *p* = 0.003). There was no difference in uptake in the reference muscle tissue. (**D**) [^64^Cu]NODAGA-cetuximab uptake on day 10 of the study at 6 h, 24 h and 48 h after injection. No differences were found between the groups at any timepoint. Data are shown as mean ± standard deviation (SD).

The A431 tumors of the vehicle-treated (VEC) mice revealed a significant increase in tumor volume over the 12 days of the study (*p* = 0.00006). Figure 4A shows the development of tumor volumes over the course of the study. Although the tumors of the genistein-treated (GEN) group showed a reduced tumor growth, significance was only reached in the cetuximab-treated (CET) and the genistein-cetuximab combination-treated (GEN-CET) groups compared to the VEC group (*p* = 0.0003 and *p* = 0.0005, respectively). However, there was no significant difference in tumor growth between the CET and the GEN-CET groups.

#### PET imaging

On day 5 of the study the tracer [^18^F]FLT was used to monitor the treatment progression (Figure 4B). The VEC group showed the lowest [^18^F]FLT uptake (mean %ID/cc 8.35 ± 1.11). The GEN as well as the CET groups revealed significantly higher %ID/cc mean values, 9.96 ± 1.36 (*p* = 0.04) and 10.54 ± 1.75 (*p* = 0.03), respectively. The tumor uptake of the GEN-CET animals (12.90 ± 1.21 %ID/cc) was the highest with significant differences to the VEC as well as the GEN groups (*p* = 0.0002 and *p* = 0.01, respectively) but not to the CET group.

The tracer uptake of the liver showed that the therapies influenced the liver tissue in a significant manner. Other than the tumors the liver tissue of the CET group revealed the lowest tracer uptake (%ID/cc 1.40 ± 0.38) whereas the uptake of liver tissue of the GEN and the GEN-CET groups was significantly higher (2.50 ± 0.30, *p* = 0.0001, and 2.53 ± 0.14, *p* = 0.00007, respectively). Although the tracer uptake in liver tissue (%ID/cc 2.06 ± 0.42) of the VEC group was significantly lower compared to that of the GEN (*p* = 0.03) and the GEN-CET groups (*p* = 0.02) it was still higher than the uptake of the CET group liver tissue.

On day 8 of the study, [^18^F]FDG was administered and all mice of the four groups were measured in PET (Figure 4C). The VEC group showed a mean %ID/cc of 2.71 ± 0.57. All three treatment groups revealed a significantly higher tracer uptake compared to the VEC group. The GEN group showed a mean %ID/cc of 3.37 ± 0.25 (*p* = 0.008), the CET group of 3.48 ± 0.30 (*p* = 0.004) and the GEN-CET group of 3.56 ± 0.19 (*p* = 0.003). Among the treated groups no significant differences were found. The muscle tissue was set as reference and showed no significant differences in the uptake between the groups (VEC: 1.14 ± 0.19, GEN: 1.09 ± 0.19, CET: 1.31 ± 0.25 GEN-CET: 0.97 ± 0.32).

[^64^Cu]NODAGA-cetuximab imaging was performed on days 10-12 of the study with measurements 6 h, 24 h and 48 h after tracer injection (Figure 4D). No significant differences in tumor tracer uptake between the 4 groups were obtained at any time point. Figure S3 presents the tracer uptake in the liver and muscle tissue.

An EGFR Western Blot analysis was performed to determine the EGFR expression level which is shown in the supplementary data (Figure S4). By visual inspection only minor differences in the EGFR levels were found revealing no clear reduction during treatment.

#### HR-MAS NMR metabolic fingerprinting

**Figure 5:**
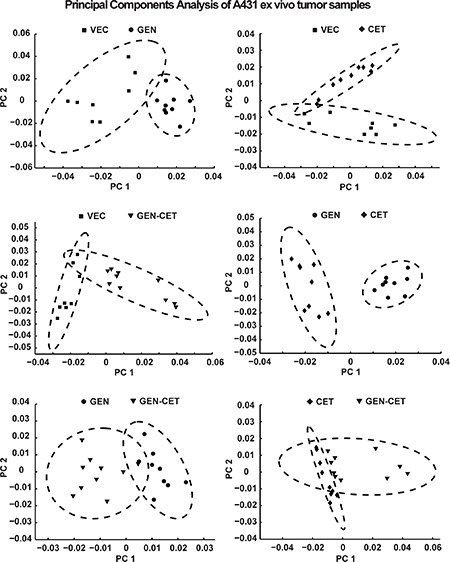
Scores plot of the first and second principle components from the pair-wise PCA of the A431 tumor HR-MAS NMR spectra of the four different treatment groups While a separation can be seen in the first principle component between vehicle-treated (VEC) and genistein-treated (GEN), VEC and genistein-cetuximab-treated (GEN-CET), GEN and cetuximab-treated (CET) as well as GEN and GEN-CET groups as in the second principle component for VEC and CET groups, no clear separation is found between CET and GEN-CET groups.

Figure 5 shows the results of the first and second principal components of the pair-wise PCA in a scores plot. A high variation is present in the spectral patterns within the VEC, CET and GEN-CET groups. Nevertheless, a separation due to extensive metabolic differences in the NMR spectra is present between VEC and GEN, GEN and CET as well as VEC and CET (in PC 2), and also between VEC and GEN-CET as well as GEN and GEN-CET. No clear separation was found between CET and GEN-CET.

## DISCUSSION

Genistein is a phytoestrogen and subject in an increasing number of publications over the past 10 years. As a naturally occurring isoflavone the research interests concentrate on its pharmacological activities as a tyrosine kinase inhibitor, its phytoestrogen activity or its chemoprotectant properties [1, 2, 6, 12]. A great number of interactions of genistein with cellular pathways have been investigated so far [12, 13]. We investigated genistein therapy in two different xenograft mouse models with three different PET tracers to identify a feasible PET tracer for therapy monitoring on the molecular level.

In both xenograft models, A431 and Colo205, a daily, per orally given therapy with 500 mg/kg genistein over a study period of 12 days was highly effective. Genistein administration resulted in significantly decreased tumor growth compared to that in vehicle-treated mice.

When [^18^F]FDG was used, the A431 xenografts treated with genistein exhibited a significantly higher tracer uptake compared to the vehicles on both imaging days. The Colo205 xenografts revealed no significant differences in [^18^F]FDG uptake between all groups on both measurement days. We performed not only histology of the tumor tissue but also cell culture experiments to evaluate, if the *in vitro* results would be consistent with *in vivo* findings. Furthermore, we were interested to see whether the kinase inhibitory effects of the phytoestrogen genistein would alter [^18^F]FDG uptake differently compared with the synthetic estrogen ethinylestradiol modulating only ERα and ERβ activity. The amount of orally administered genistein that is available *in vivo* is in accordance with *in vitro* concentrations between 50 μM and 100 μM using the studies of bioavailability and pharmacokinetics of genistein by Yang et al. [24]. Interestingly, when A431 cells were incubated with 100 μM genistein the tracer uptake was also significantly increased compared to the control cells after 48 h and 72 h, respectively. Although the GLUT1 index did not reveal significant differences between treated and control tumor tissue, the GLUT3 level was significantly elevated in genistein-treated mice. There is evidence that genistein inhibits GLUT1 and GLUT4 on a micromolar level [25, 26] whereas it was shown by others that the isoflavone could reverse the GLUT3 downregulation in ovariectomized rats [27]. Since we found a significantly higher [^18^F]FDG uptake when genistein treatment was used *in vivo* as well as *in vitro* and histology revealed a significantly higher GLUT3 index, we could hypothesize that genistein up-regulates the expression of GLUT3 in A431 cells which can be indirectly monitored by [^18^F]FDG. The cell culture experiments performed with ethinylestradiol revealed that [^18^F]FDG uptake increased with higher concentrations and longer incubation times but in a less strong fashion compared to genistein-treated cells. Zhao et al. reported in an imaging study of patients with mesenchymal uterine tumors a negative correlation between [^18^F]FDG uptake and ERα and a positive correlation with ERβ [28]. It was also shown that genistein inhibits the proliferation and differentiation of human breast cancer cells through down-regulation of ERα [29]. Therefore it is likely that genistein treatment in A431 xenografts might also provoke down-regulation of ERα while up-regulating GLUT3 at the same time. The [^18^F]FDG uptake in genistein-treated Colo205 cells was significantly lower with high concentrations of genistein compared to the control cells at later timepoints. When ethinylestradiol was used, there was also significantly less [^18^F]FDG uptake with high concentrations at later timepoints compared to control cells but compared to genistein treated cells the tracer uptake revealed to be much higher. In contrast to A431 cells Colo205 cells lack ERα and only express ERβ [30] which could play a role in the difference of tracer uptake between the two cell lines. In the cell culture experiments we could show that inhibitory differences exist between treatment with genistein and ethinylestradiol, respectively, which could be imaged using [^18^F]FDG. Therefore, genistein therapy clearly affects pathways beyond the two estrogen receptors.

Other than this, there was no significant difference in [^18^F]FDG uptake between the genistein-treated and the vehicle-treated Colo205 xenografts. Histology showed that GLUT3 levels were not altered but the GLUT1 index in genistein-treated tumor tissue revealed to be significantly increased compared to the vehicle group. Although GLUT1 expression is stimulated, it is also inhibited by genistein and, therefore, apparently not effecting the [^18^F] FDG uptake.

In our study, the [^18^F]FLT uptake in tumors of genistein-treated animals increased significantly compared to tumors of vehicle-treated animals, both in A431 and Colo205 *in vivo* models. Consistently, TK1 indices were significantly increased in the tumor tissue of both xenograft models when genistein therapy was applied. [^18^F]FLT uptake mainly mirrors the TK1 expression level while other histological markers for proliferation, like Ki67, often yield discrepant results when they are used for correlation to the tracer uptake [31]. TK1 is typically closely correlated with the DNA synthesis (late G1/S phase) of proliferating cells [18]. Until now, the only study where TK1 was evaluated in combination with genistein treatment was performed in irradiated myelogenous leukemia cells [32]. Jeong et al. found that genistein treatment induced G2/M phase cell cycle arrest after G1/S progression leading to an upregulation of TK1 enzymatic activity before cells went into apoptosis [32]. We hypothesize that in both A431 and Colo205 xenograft models genistein-treatment exhibited this effect supported by [^18^F]FLT uptake and histological findings assigning [^18^F]FLT as suitable tracer to monitor genistein therapy.

In a separate study we investigated the effect of the EGFR specific mAb cetuximab (CET) on the A431 xenograft tumor growth as well as the combination therapy of cetuximab and genistein (GEN-CET). Since genistein also inhibits the EGFR we were interested to see if the two inhibitors exhibit a potentiating effect which could be monitored by PET tracer uptake in A431 tumors that highly express EGFR. The tumor growth was significantly reduced using cetuximab treatment but there was no obvious additive effect of the two treatments in inhibition of tumor growth after 12 days. [^18^F]FDG imaging on day 8 showed that the uptake of the tracer in all treatment groups was significantly increased compared to the vehicle group. Using the tracer [^18^F]FDG we found no significant differences in tumor uptake between treatment groups indicating that [^18^F]FDG is not able to differentiate the effects of the small molecular inhibitor and the mAb cetuximab. Others found that in colorectal tumor cells cetuximab treatment increased hexokinase activity which could lead to an increase in [^18^F]FDG uptake in the A431 tumor model [33]. When [^18^F]FLT was used in our study on day 5, tumor bearing GEN-CET mice exhibited a significantly higher tracer uptake compared to VEC and GEN groups potentially revealing a different effect on the molecular level of the combination therapy compared to the single treatments. Since cetuximab inhibits not only angiogenesis and induces apoptosis, it also induces G1 cell cycle arrest in A431 cells and, therefore, TK1 up regulation is followed by enhanced tracer uptake [34]. Both, genistein and cetuximab, induce cell cycle arrest after accelerated TK1 activation [32, 34]. The observed potentiated increase in [^18^F]FLT tracer uptake in tumors of GEN-CET mice could be the response to the accelerated TK1 activation.

The [^64^Cu]NODAGA-cetuximab imaging did not reveal significant differences between the groups. Nevertheless, the A431 tumor is fast-growing in our model and according to the literature exhibits a high level of angiogenesis developing an immature vasculature with the majority of vessels being small and scarcely branched [35]. In case of the antibody-based tracer [^64^Cu] NODAGA-cetuximab we could hypothesize that this might lead to an hindered distribution throughout the tumor tissue and, thus, to a decreased tissue uptake.

The pair-wise PCA of the A431 tumor HR-MAS NMR fingerprint spectra revealed distinct changes in the metabolic profile of the four different treatment groups clearly separating the groups. Metabolic fingerprinting via HR-MAS NMR has the potential to detect and characterize alterations to cellular metabolism of tumor tissue that are important hallmarks of cancer. This method can directly report the metabolome of healthy or diseased tissues, where biomarker variations are expected to be crucial, and can be used as cancer phenotyping at different stages. NMR based fingerprinting is seen as near-real-time surgical investigation because tissue samples can be analyzed in several minutes without any further preparation [36]. In our study, treatment-related differences in NMR fingerprint spectra could be explicitly detected between all pairs except CET and GEN-CET. Here, the changes in the metabolic profiles might be too small for a clear separation. Nevertheless, all other groupwise comparisons revealed extensive metabolic changes in NMR-based fingerprinting.

In conclusion, we showed that in the xenograft tumor mouse models A431 and Colo205 the isoflavone genistein is effective in antitumor therapy. Although genistein is often used in combination with synthetic drugs or therapeutic antibodies, we demonstrated a tumor reducing effect when used in monotherapy. Our aim was to monitor the therapy effects using three different PET tracers [^18^F]FDG, [^18^F]FLT and the antibody based tracer [^64^Cu]NODAGA-cetuximab as well as *ex vivo* histology and NMR metabolomic fingerprinting. We found that genistein-mediated effects on [^18^F]FLT uptake were consistent in both xenograft models and corresponded to the histological findings. Therefore, [^18^F]FLT can be used as tracer for therapy monitoring. Additionally, the combined antitumoral effects of genistein with the mAb cetuximab could be shown using this tracer. The PCA of the NMR fingerprint spectra showed significant differences in the metabolic spectrum of the genistein-treated tumors not only in comparison to vehicle-treated but also to cetuximab-treated tumors suggesting a completely different mode of action of the two therapeutics. Genistein revealed its potency in cancer therapy in our studies and our results further indicate that treatment effects can be monitored non-invasively with the PET proliferation marker [^18^F]FLT.

## MATERIALS AND METHODS

### Cell culture experiments

A431 (ATCC) cells were cultured in RPMI 1640 medium (Biochrom AG, Berlin, Germany) supplemented with 10% fetal calf serum (Biochrom AG) and penicillin/streptomycin (1% (v/v), Biochrom AG) and maintained at 37°C in a humid atmosphere with 5% CO_2_. Cells were split twice a week for three to four weeks before the experiments ensuring a high cell density. Colo205 (ATCC) cells were also cultured in modified RMPI 1640 medium under the same conditions as A431 cells.

Genistein was purchased from Alfa Aesar (Karlsruhe, Germany), DMSO from Carl Roth (Karlsruhe, Germany) and ethinylestradiol from Sigma Aldrich (Munich, Germany). Stock solutions of genistein or ethinylestradiol in DMSO were prepared for further dilutions in phosphate-buffered saline (Dulbecco's PBS, PAA Laboratories, Pasching, Austria) to reach a final concentration of DMSO in the culture media below 0.1%. The experimental setup for the cell culture experiments was identical for both cell lines and is based on cell culture experiments using the two cell lines as well as genistein or ethinylestradiol, respectively [30, 37, 38]. Cells were seeded at a density of 0.5*10^6^ cells/well into 24 well plates and 1.5 mL treatment medium was added starting drug treatment directly at the seeding time point. Treatment medium consisted of the growth medium and either genistein to reach final concentrations of 100 μM, 50 μM and 1 μM or ethinylestradiol to reach final concentrations of 50 nM, 10 nM or 5 nM. DMSO was added into the growth medium of control cells to ensure that cell viability and proliferation was not influenced. All treatments and controls were performed in triplicates. In total 4 experiments were performed using the described treatment scheme and cells were incubated for 12 h, 24 h, 48 h or 72 h, respectively. After the incubation time 1.85 MBq of [^18^F]FDG was added to each well. At the end of the 1 h uptake time treatment medium was removed and cells were washed twice with cold PBS. The cells were harvested in 1 mL of lysis buffer (125 mM NaCl, 25 mM HEPES, 4.8 mM KCl, 1.2 mM KH_2_PO_4_, 1.2 mM CaCl_2_, 1.2 mM MgSO_4_, 5.6 mM glucose, pH 7.4 and 0.2% sodium dodecyl sulfate) and transferred into gamma counter tubes. A gamma counter standard was prepared using 5 × 1 mL from a 3.7MBq [^18^F]FDG in 50 mL water solution. After gamma counting the cell suspension was frozen at −20°C and a protein assay was performed using the Micro BCA protein assay kit (Thermo Scientific, Rockford, Illinois, USA) to determine the protein concentration of the treated and control cells. The assay was performed following the manufacturer's instructions. The [^18^F]FDG uptake was calculated as % incubated dose per milligram protein (%ID/mg).

### Mouse xenograft model

Female NMRI nu/nu mice (6–7 weeks, 20–25 g) were purchased from Charles River Laboratories, St. Germain Sur L'Arbresle, France, and received water and food ad libitum. They were kept under standardized conditions with 50 ± 10% relative humidity, 20 ± 1°C and a 12 h light-dark cycle. All animal experiments were approved by the governmental administrations of Tuebingen, Germany, and in accordance with the German Animal Protection Law.

On the day of inoculation A431 and Colo-205 cells, respectively, were trypsinized, washed twice with PBS and counted using a Neubauer chamber. 5*10^6^ A431 cells or 3*10^6^ Colo-205 cells in a volume of 200 μL PBS solution were inoculated subcutaneously into the upper right flank of the animals, respectively, while they were anaesthetized with 1.5% isoflurane vaporized in 0.8 L/min 100% oxygen.

9 days after inoculation tumors reached sizes of approximately 0.2 cubic centimeter (cc) and treatment was started after randomization of the animals. Tumor volumes were measured using calipers and calculated according to the equation: Volume[cc] = π*a*b^2^/6000, where a was the long and b the short axis.

### Drug treatment

In the first part of the study, a total of 40 animals were used, 20 mice bearing A431 and 20 mice Colo205 tumors. After randomization 10 animals of the A431 tumor bearing group represented the vehicle group (VEC-A431) and 10 animals were treated with 500 mg/kg genistein daily per oral (GEN-A431) for a total of 12 days. The solution was prepared freshly every day using a mixture of 70% PBS, 15% ethanol (Merck, Darmstadt, Germany) and 15% cremophor EL (Sigma-Aldrich, Munich, Germany). Vehicle mice (VEC) received the solution without genistein daily per oral. The same treatment was performed for the Colo-205 bearing mice (10 animals represented the VEC-Colo-205 group and 10 the GEN-Colo-205 group). After 12 days of treatment the animals were sacrificed for histology.

In the second part of the study, only A431 tumor bearing mice were included. A total of 20 mice were divided and randomized into 4 groups. One group of animals represented the vehicle group (VEC). From the first day of treatment one group of animals (GEN) received the genistein solution (500 mg/kg/d per orally). One group of animals (CET) was medicated with 1 mg cetuximab intraperitoneally (Merck Serono, Darmstadt, Germany) in its clinically available form every third day of the study. A combination of cetuximab and genistein was administered to the fourth group of animals (GEN-CET). After 12 days of treatment the animals were sacrificed for *ex vivo* NMR-based metabolic fingerprinting.

### Production of radiopharmaceuticals

To produce [^18^F]fluoride the ^18^O(p,n)^18^F nuclear reaction was performed using the PETtrace cyclotron (General Electric Medical Systems, GEMS, Uppsala, Sweden). Syntheses of [^18^F]FDG and [^18^F]FLT were described elsewhere [39, 40]. The specific activities were calculated at the end of synthesis and revealed > 50 GBq/μmol for [^18^F]FDG and 100 – 200 GBq/μmol for [^18^F]FLT with radiochemical purity of > 99%.

For the [^64^Cu]-NODAGA-cetuximab synthesis (see Supplementary Section for further details), 500 MBq of freshly prepared ^64^Cu and 480 μg of NODAGA-cetuximab were used. The ^64^Cu solution (∼80 μL) was adjusted to pH 7.0 by adding 10x PBS (∼8 μL, Carl Roth) before the NODAGA-cetuximab solution (58 μL) was added. The mixture was incubated for 1h at 42°C before quality control was performed using TLC (silica gel, Macherey-Nagel, Polygram SilG/UV_254_) with 0.1 M sodium citrate (pH 5) as mobile phase. Subsequent detection of the radiotracer was performed using a Cyclone Plus PhosphorImager (Perkin-Elmer). The radiochemical purity was assessed with 94%.

### Small animal PET and anatomical MRI measurements

PET imaging was performed on two identical small animal PET scanners (Inveon, Siemens Preclinical Solutions, Knoxville, TN, USA), yielding a 1.4 mm spatial resolution in the reconstructed image [41]. PET images were reconstructed using an iterative two-dimensional ordered subset expectation maximization algorithm (OSEM2D). Attenuation correction was not applied and normalization was performed according to the injected activity.

Before PET tracer injection mice were anaesthetized with 1.5% isoflurane vaporized in 0.8 L/min 100% O_2_ in a 37°C heated anesthesia chamber. Prior to [^18^F]FDG measurements, mice were fasted for 12 h, and body weight and tumor volume were measured. Before [^18^F]FDG injections blood samples were taken from each mouse by retrobulbar puncture for blood glucose measurements using a HemoCue glucose system (HemoCue GmbH, Grossostheim, Germany). Static [^18^F]FDG and [^18^F]FLT scans were performed for 10 min after an uptake period of 55 min or 90 min, respectively. Following [^18^F]FLT injection, mice were awake during the uptake period. Immediately after injection of [^64^Cu]NODAGA-cetuximab, mice were returned to their cages and kept conscious during uptake. They were scanned at 6 h, 24 h, and 48 h after tracer injection. For all tracer injections approximately 13 MBq were administered intravenously via the tail vein.

After the PET scan half of the mice were transferred on the same bed under anesthesia to the 7T MRI system (ClinScan, Bruker, Ettlingen, Germany) to acquire an anatomical scan for the unambiguous analysis of the PET images. Anatomy measurements were performed using the turbo spin echo sequence (TE = 205 ms, TR = 3000 ms, Resolution: 0.2 × 0.2 × 0.2 mm³).

For the first part of the study, all mice were measured with [^18^F]FDG on days 8 and 12 and with [^18^F]FLT on days 5 and 11 of the study. Immediately after the last PET or MRI scan, respectively, on day 12 of the study mice were sacrificed and several organs including tumor, blood and muscle tissue were excised for gamma counting. For the second part of the study, all mice were measured with [^18^F]FLT on day 5, with [^18^F]FDG on day 8 and with [^64^Cu]NODAGA-cetuximab consecutively on days 10 to 12 before the animals were sacrificed immediately after the last PET scan on day 12. Tumor tissue was taken and instantly frozen in liquid nitrogen for HR-MAS NMR spectroscopy.

For the analysis of the PET scans the available MR images were manually co-registered using the Inveon Research Workplace (Siemens Preclinical Solutions) software. Regions of interest (ROIs) were drawn on coronal PET images by visual inspection using the MR images as tissue references to obtain the tumor tracer uptake in the PET images (Bq/mL). Quantification of PET data was expressed as mean % injected dose per cubic centimeter (%ID/cc) for each animal.

### Histology and immunohistochemistry

In the first part of the study, histology and immunohistochemistry was performed after the gamma counting. Tumors were fixed in 4.5% buffered formalin for 24 h before embedding in paraffin for histological staining. 3–5 μm sections were cut and stained with haematoxylin and eosin (H & E). Immunohistochemistry was performed on an automated immunostainer (Ventana Medical Systems, Inc.) according to the manufacturer's protocols for open procedures. Slides of every tumor were stained with Ki67 antibody (SP6, DCS Innovative Diagnostik-Systeme GmbH u. Co. KG, Hamburg, Germany), Cleaved Caspase 3 (ASP 175; Cell Signaling Technology, Frankfurt am Main, Germany), TK1 (Abcam plc, 330 Cambridge Science Park, Cambridge, UK), GLUT1, GLUT3 and GLUT4 (Abcam Inc., Suite B2304 Cambridge, USA). Appropriate positive and negative controls were used to confirm the adequacy of the staining. The indices of Ki67 and cleaved Caspase 3 tumor sections were evaluated by counting 100 cells in three different areas per tumor. GLUT1, GLUT3, GLUT4 and TK1 indices were determined using the high power field (400 point magnification). The H & E results show the mean % necrosis of the tumor tissue, the immunohistochemistry results show the mean % positive cells within viable tumor regions.

### Metabolite fingerprinting using high resolution magic angle spinning nuclear magnetic resonance spectroscopy (HR-MAS NMR)

In the second part of the study, mice were sacrificed tumors were taken out, weighted and frozen in liquid nitrogen. Tumors were stored at −80°C until HR-MAS sample preparation. Sample preparation was done using the −20°C environment of a cryotome. Approximately 10 mg of tumor tissue were cut using biopsy punches (Calmed, Wesel, Germany) and inserted into a 33 μL disposable insert made of Kel-F (Bruker, Karlsruhe, Germany). 10 μL of D_2_O were added to the tumor sample and the insert was closed with a cap. Two samples were prepared for each tumor yielding 40 NMR samples. Several tumor samples were used for EGFR Western Blot analysis after preparation of the NMR samples. Spectra were recorded on a 600 MHz Bruker Avance III spectrometer equipped with a HR-MAS probe (Bruker Biospin, Wissembourg, France) at 277 K and a rotation speed of 5,000 Hz. ^1^D-^1^H CPMG (Carr-Purcell-Meiboom-Gill) spectra with water presaturation were recorded with the following parameters: ns = 128, 15 ppm sweep width, 1.81 sec acquisition time, loop 320 and a time domain of 32 k.

Spectra were post-processed using Topspin 3.2. Three spectra (2 spectra from the VEC and 1 spectrum from the GEN and CET groups, respectively) had to be discarded because of high lipid content. For principal component analysis (PCA), the remaining spectra were further processed using MATLAB (The MathWorks Inc, Natick, MA). Spectra were aligned using icoshift [42] and binned into equi-distant frames of 0.03 ppm. Water and lipid regions were excluded and the spectra were scaled to the signal integral of the remaining regions. PCA was performed using MATLAB without additional scaling or weighting.

### Statistics

It was ensured for all data that the required criteria were met before any statistical tests were applied. Origin 8.0 Pro (Origin LabCorporation, Northampton, MA, USA) was applied for multigroup comparisons using a one way analysis of variance (ANOVA) and all *p*-values were subsequently corrected followed by Bonferroni Multiple Comparison Test.

Data homoscedasticity was tested applying the Brown-Forsythe test. For the comparison of two groups or different days within each group, a two sided *t*-test was used. Statistical significance was considered for *p* < 0.05 (*) and *p* < 0.001 (**). All quantitative results are shown as mean ± standard deviation (SD).

## SUPPLEMENTARY MATERIALS FIGURES


