# Supplementary material for: Preclinical evaluation of the anti-tumor effects of the natural isoflavone genistein in two xenograft mouse models monitored by [18F]FDG, [18F]FLT, and [64Cu]NODAGA-cetuximab small animal PET
Source: Oncotarget. 2016 Apr 7;7(19):28247–61. doi: 10.18632/oncotarget.8625 (PMC5053724; doi:10.18632/oncotarget.8625)
Supplement: Supplementary file 1 [file oncotarget-07-28247-s001.pdf]

Preclinical evaluation of the anti-tumor effects of the natural isoflavone genistein in two xenograft mouse models monitored by [^{18}F]FDG, [^{18}F]FLT, and [^{64}Cu]NODAGA-cetuximab small animal PET

Supplementary Materials

RESULTS

Animal Weight

Over the course of the treatment animal weight was monitored to assure that the animals did not lose weight as side effect of the treatment. Figure S1A and S1B show the animal weight of the A431 and Colo205 xenografts for genistein treated as well as vehicles over the study period of 12 days. Figure S1C depicts the animal weight of A431 xenografts when not only genistein was used as treatment but also cetuximab and the combination therapy of genistein and cetuximab. The animal weight was constant over the whole study period. Neither treatment caused any weight loss or other visible side effects.

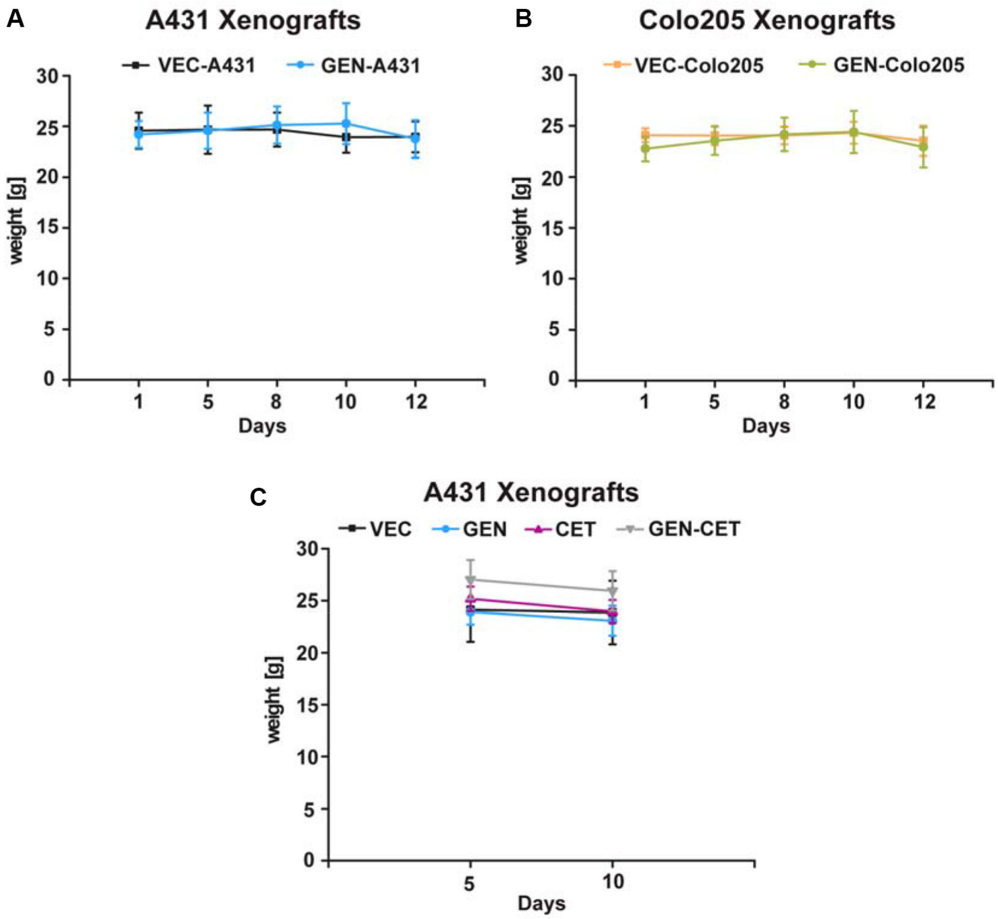

Supplementary Figure S1: animal weight of all animals used in this study. (**A**) animal weight of A431 xenografts, vehicle-treated (VEC-A431) and treated with genistein (GEN-A431); (**B**) animal weight of Colo205 xenografts, vehicle-treated (VEC-Colo205) and treated with genistein (GEN-Colo205); (**C**) animal weight of A431 xenografts, vehicle-treated (VEC) and treated with genistein (GEN), treated with cetuximab (CET) and a combination treatment of genistein and cetuximab (GEN-CET).

Genistein Treatment in A431 and Colo205 Xenografts

PET Imaging

Figure S2A shows the liver tissue uptake of the proliferation marker [^{18}F]FLT on day 5 (VEC-A431 1.80 ± 0.36 and GEN-A431 2.12 ± 0.26, $p = 0.03$) and on day 11 (VEC-A431 1.78 ± 0.29 and GEN-A431 2.32 ± 0.33, $p = 0.001$) for the A431 xenografts as well as for the Colo205 xenografts (on day 5: VEC-Colo205 1.88 ± 0.36 and GEN-Colo205 2.37 ± 0.53, $p = 0.03$ and on day 11: VEC-Colo205 1.67 ± 0.23 and GEN-Colo205 2.79 ± 0.55, $p = 0.00002$). The tracer uptake is significantly different between treated and untreated animals.

Figure S2B shows the muscle uptake of the glucose analogon [^{18}F]FDG which revealed no changes over time (VEC-A431 1.05 ± 0.20 on day 8 and 1.01 ± 0.20 on day 12, GEN-A431 1.02 ± 0.19 on day 8 and 1.09 ± 0.20 on day 12) for the A431 xenografts as well as for the Colo205 xenografts (VEC-Colo205 0.97 ± 0.19 on day 8 and 1.07 ± 0.25 on day 12, GEN-Colo205 0.93 ± 0.18 on day 8 and 0.99 ± 0.11 on day 12).

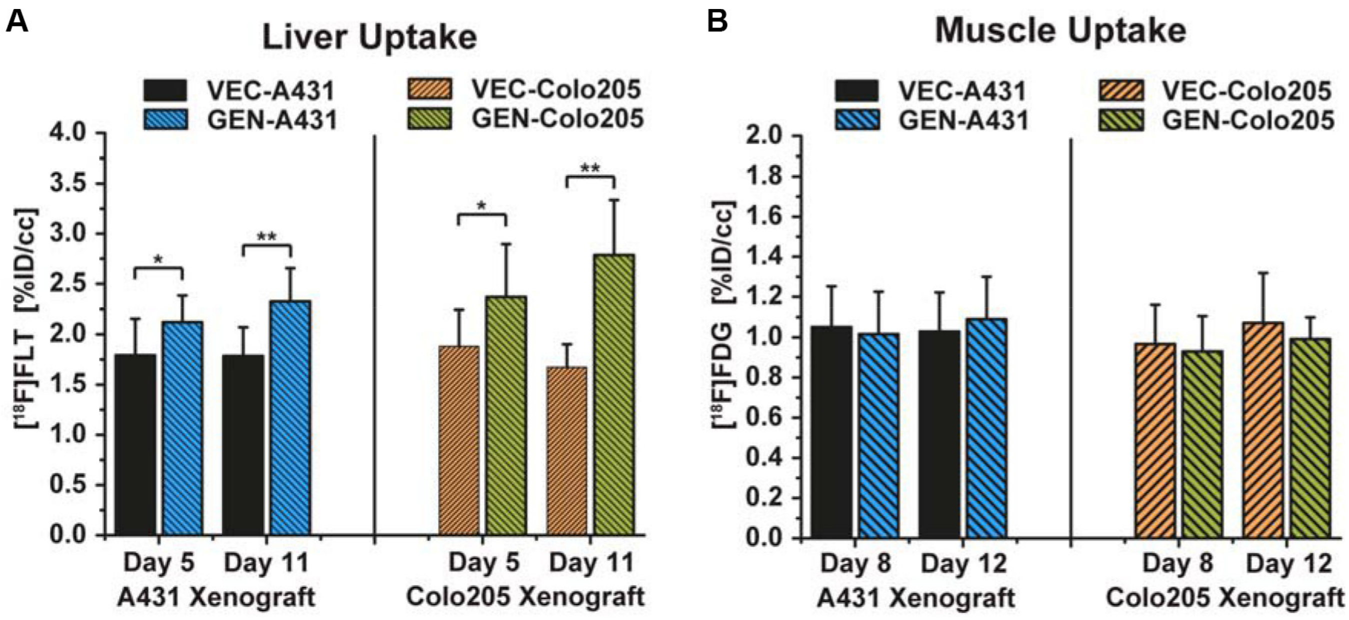

Supplementary Figure S2: (**A**) [^{18}F]FLT uptake of the reference tissue liver of both xenograft models on day 5 and 11 of the study, both xenografts revealed significantly higher tracer uptake when the mice were treated with genistein (GEN-A431 and GEN-Colo205, respectively) compared to the vehicle-treated groups (VEC-A431 and VEC-Colo205, respectively) on both imaging days, (**B**) [^{18}F]FDG uptake of the reference tissue muscle of both xenograft models on day 8 and 12 of the study, there were no significant differences between the genistein-treated (GEN-A431 and GEN-Colo205, respectively) and the vehicle-treated (VEC-A431 and VEC-Colo205, respectively) groups. Data are shown as mean ± standard deviation (SD).

Comparison of genistein, cetuximab and combined genistein-cetuximab treatments in A431 xenografts

[^{64}Cu]NODAGA-cetuximab imaging was performed on days 10–12 of the study with measurements 6 h, 24 h and 48 h after tracer injection (Figure 4D). No significant differences in tracer uptake between the 4 groups were obtained at any time point. Figure S3 presents the tracer uptake in the liver and muscle tissue. The racer uptake in the liver after 24 h of injection shows a significantly lower uptake in the GEN-CET group compared to the vehicle. There is no such finding in the tracer uptake of the muscle over time.

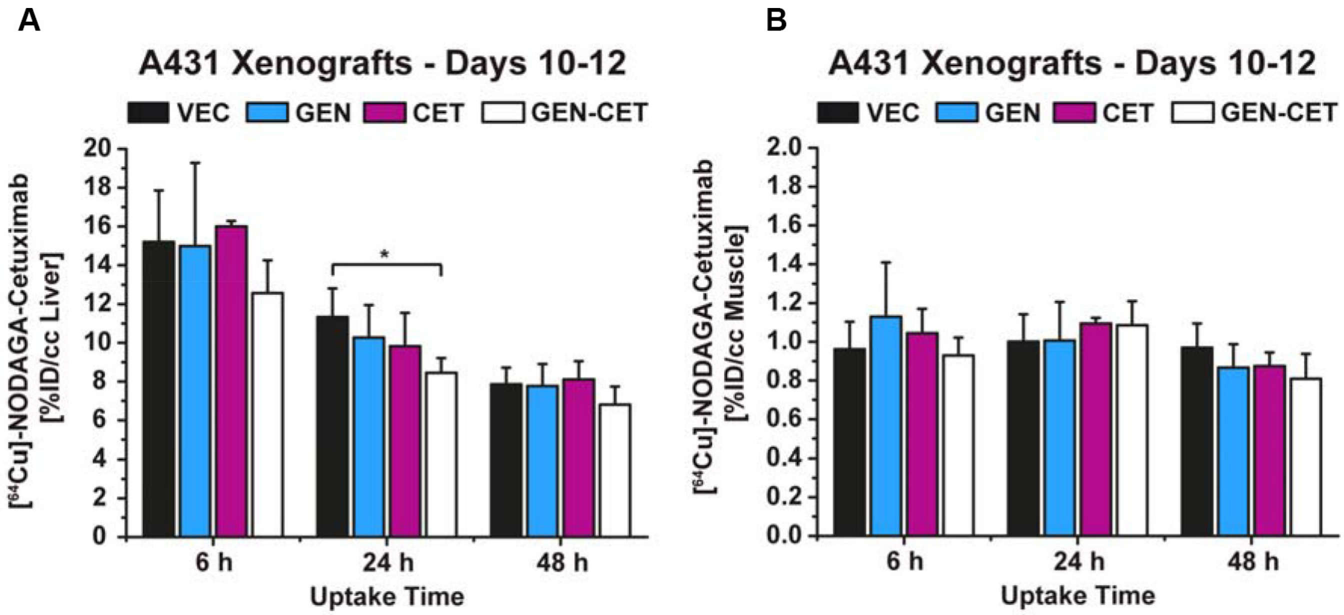

Supplementary Figure S3: [^{64}Cu]NODAGA-cetuximab uptake of the 4 groups on day 10 of the study at 6 h, 24 h and 48 h after injection for (A) the liver tissue and (B) the muscle tissue. Whereas the muscle tissue did not show any differences the liver tissue revealed a significantly lower uptake in the GEN-CET group compared to the VEC group at 24 h after injection. Data are shown as mean ± standard deviation (SD).

EGFR Western Blot

Figure S4 shows the Western Blot analysis of tumors of the VEC, GEN, CET, and GEN-CET groups. In the treatment groups only a slight reduction of EGFR was found compared to the VEC group.

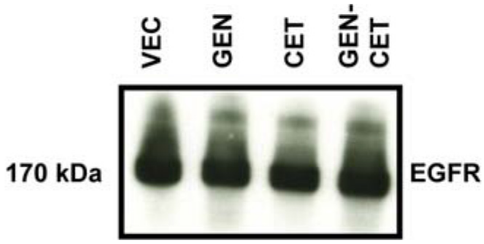

Supplementary Figure S4: Epidermal growth factor receptor (EGFR) Western Blot analysis of tumors of the four different treatment groups to reveal the level of EGFR in the tumors.

MATERIALS AND METHODS

Synthesis of NODAGA-cetuximab

For NODAGA conjugation and radiolabeling of cetuximab, NODAGA NHS ester was obtained from Chematech (Dijon, France) and conjugated to cetuximab (Erbitux, Merck, Darmstadt, Germany) according to published methods [1, 2]. 1 mL of cetuximab solution (5mg/ml) was diafiltrated against Na+Chelex 100 treated (Sigma Aldrich, Munich, Germany) 0.1M sodium phosphate buffer pH 7.5 using Amicon Ultra-15 centrifugal filter units (MWCO 30 kDa, Merck). NODAGA NHS ester was dissolved in ultrapure water (Rotipuran, Carl Roth, Karlsruhe, Germany) and immediately added to the antibody at a molar ratio of 55:1. After incubation over night at 4 °C the chelator excess was removed and the protein was concentrated by repeated diafiltration with D-PBS (Gibco, Life Technologies, Carlsbad, CA, USA) using Amicon Ultra-15 filter units (MWCO 30 kDa). A NanoDrop 1000 spectrophotometer (Peqlab, Erlangen, Germany) was used for measuring OD280 nm and calculating the protein concentration.

^{64}Cu was produced via the nuclear reaction of ^{64}Ni(p, n)^{64}Cu using a PETtrace cyclotron (GE Healthcare, Uppsala, Sweden) at 12.4 MeV as described by McCarthy et al. [3] and separated from Ni and other impurities via ion exchange chromatography, trapping ^{64}Cu while eluting ^{64}Ni. Briefly, ^{64}Ni (20 mg, > 98% enrichment) was electroplated on a platinum/iridium plate (90/10) and irradiated to generate [^{64}Cu]Ni. The target was dissolved in 3 ml concentrated HCl and evaporated to dryness. The residue was dissolved in 4% 0.2 N HCl/ 96% (v/v) methanol and passed through the ion exchange column (AG1-X8 column, BioRad). ^{64}Cu was released from the column using 0.9M HCl containing 30% (v/v) isopropanol. After complete evaporation 64Cu was dissolved in 140–210 µL of 0.1N HCl.

Western blot for EGFR detection

Several tumor samples were used for EGFR Western Blot analysis after preparation of the NMR samples. Frozen tumor tissue samples were extracted using lysis buffer to estimate the levels of EGFR in the different treatment groups. After centrifugation equal amounts of supernatant were separated by SDS-PAGE and electrophoretically transferred to PVDF membranes. 5 µg of the supernatant were loaded onto the gel. Membranes were blocked in 5% nonfat milk in TBS/0.1% Tween20 (Sigma-Aldrich) and incubated with anti-EGFR (1:1000, rabbit, Santa Cruz Biotech) and anti-GAPDH (1:5000, mouse, Millipore). Secondary antibodies from Jackson Immunoresearch were applied (1:15000) and protein bands were visualized using ECL plus (GE Healthcare).

REFERENCES

1. Elsasser-Beile U, Reischl G, Wiehr S, Buhler P, Wolf P, Alt K, Shively J, Judenhofer MS, Machulla HJ, Pichler BJ. PET imaging of prostate cancer xenografts with a highly specific antibody against the prostate-specific membrane antigen. J Nucl Med. 2009; 50:606–611.
2. Griessinger CM, Maurer A, Kesenheimer C, Kehlbach R, Reischl G, Ehrlichmann W, Bukala D, Harant M, Cay F, Bruck J, Nordin R, Kohlhofer U, Rammensee HG, et al. 64Cu antibody-targeting of the T-cell receptor and subsequent internalization enables *in vivo* tracking of lymphocytes by PET. Proc Natl Acad Sci U S A. 2015; 112:1161–1166.
3. McCarthy DW, Shefer RE, Klinkowstein RE, Bass LA, Margeneau WH, Cutler CS, Anderson CJ, Welch MJ. Efficient production of high specific activity 64Cu using a biomedical cyclotron. Nucl Med Biol. 1997; 24:35–43.
